# Efficacy of immune checkpoint inhibitors in non-small cell lung cancer with NTRK family mutations

**DOI:** 10.1186/s12890-023-02707-x

**Published:** 2023-11-29

**Authors:** Xiaoling Shang, Wengang Zhang, Wenfei Han, Handai Xia, Ni Liu, Xiuwen Wang, Yanguo Liu

**Affiliations:** Department of Medical Oncology, Qilu Hospital of Shandong University, 107 Wenhuaxi Road, Jinan, Shandong 250012 China

**Keywords:** Non-small cell lung cancer, NTRK family mutation, Immune checkpoint inhibitors, Tumor mutational burden, PD-L1 expression, Overall survival

## Abstract

**Background:**

The efficacy of immune checkpoint inhibitors (ICIs) in non-small cell lung cancer (NSCLC) patients harboring neurotrophin receptor kinase (NTRK) family mutations remains obscure.

**Methods:**

The Zehir cohort from cBioPortal was used to analyze the mutations (MT) frequency of NTRK family in patients with NSCLC, and their correlation with clinical characteristics and patient survival. The influence of NTRK MT on ICIs efficacy was evaluated in ICIs-treated patients from Samstein cohort and further validated by use of data from OAK/POPLAR cohort.

**Results:**

In the Zehir cohort, a significant difference was observed in median overall survival (mOS) between patients with NTRK MT and wild-type (WT) (mOS: 18.97 vs. 21.27 months, HR = 1.34, 95%CI 1.00-1.78; log-rank *P* = 0.047). In Samstein cohort, the mOS of NTRK mutant patients receiving ICIs has improved compared to WT patients (mOS: 21.00 vs. 11.00 months, log-rank *P* = 0.103). Notably, in subgroup analysis, ICIs significantly prolonged mOS in patients with NTRK3 MT than in WT patients (mOS: not available vs. 11.00 months, HR = 0.36, 95%CI 0.16–0.81; log-rank *P* = 0.009). Identical mOS between NTRK MT and WT patients receiving ICIs treatment (mOS: 13.24 vs. 13.50 months, log-rank *P* = 0.775) was observed in OAK/POPLAR cohort. Moreover, a similar programmed death ligand 1 (PD-L1) expression, but higher tumor mutational burden (TMB), blood TMB (bTMB) and enriched anti-tumor immunity were observed in NTRK MT compared to WT (*P* < 0.05).

**Conclusion:**

Taking high TMB or bTMB into consideration, patients with NTRK mutant NSCLC could benefit from ICIs treatment.

**Supplementary Information:**

The online version contains supplementary material available at 10.1186/s12890-023-02707-x.

## Introduction

Lung cancer remains the leading cause of cancer-related deaths worldwide, with non-small cell lung cancer (NSCLC) being the predominant pathological subtype, accounting for more than 85% [1, 2]. The majority of patients diagnosed with NSCLC are at an advanced stage, and the therapeutic paradigm for advanced NSCLC has evolved substantially over the last decade. Notably, mounting clinical trials demonstrate that immune checkpoint inhibitors (ICIs), specifically those targeting programmed death 1 (PD-1) and programmed death ligand 1 (PD-L1), have significantly increased the survival of patients with metastatic NSCLC, boosting the 5-year survival rate from less than 5% historically to approximately 30% presently for treatment-naïve patients [3, 4].

While it has been proven that ICIs are beneficial in patients without driver mutations, their efficacy in patients with driver mutations such as EGFR, ALK, or ROS1, is poor [5–7]. Two meta-analyses of clinical trials comparing anti-PD-1 or anti-PD-L1 to docetaxel demonstrated that patients with EGFR mutant NSCLC did not benefit from ICIs treatment as their wild-type counterparts [8, 9]. Additionally, results from the prospective trial showed none of the patients with EGFR mutation responded to first-line treatment with anti-PD-1 antibody pembrolizumab [10]. Relatively, low PD-L1 expression, reduced infiltration of CD8^+^ T lymphocytes and decreased immunogenicity in EGFR mutant NSCLC may be the potential mechanisms of poor response to ICIs [11, 12]. Likewise, no or minimal objective response was observed among ALK positive patients treated with ICIs [13]. Consequently, EGFR mutant or ALK positive NSCLC patients were excluded from most large clinical trials involving ICIs.

However, a subset of patients with NSCLC harboring driver mutations do respond to ICIs and experience prolonged survival, such as uncommon EGFR mutation [14]. Moreover, NSCLC patients with KRAS or BRAF mutations obtained an increased benefit from ICIs [15]. Superior survival benefit was also found in MET-altered patients compared to MET-unaltered patients subject to immunotherapy [16]. Therefore, ICIs efficacy varies according to different driver mutations, elucidating the association between driver mutation and ICIs responsiveness is crucial for therapeutic options in NSCLC, particularly in patients who initially respond poorly to targeted therapy or who have developed resistance to targeted therapy.

The neurotrophin receptor kinase (NTRK) genes family NTRK1, NTRK2 and NTRK3 encode tropomyosin receptor kinases TRKA, TRKB, TRKC, respectively [17]. NTRK fusion mutations are oncogenic drivers that have been identified in a variety of cancers including lung cancer [18]. A number of clinical trials demonstrated that TRK inhibitors (larotrectinib or entrectinib) have superior therapeutic effect compared with chemotherapy in advanced NSCLC patients with NTRK-positive mutations regardless of tumor type, fusion type and age, with a median progression-free survival (PFS) of 11.2 to 25.8 months [19, 20]. Therefore, TRK inhibitors are currently the first-line standard treatment for these patients [21, 22]. However, acquired resistance to TRK inhibitors is inevitable and innovative therapeutic strategies afterwards still remain an unresolved issue [23]. Whether ICIs can be used as a treatment option in patients with NSCLC harboring NTRK mutations have not been clarified.

In this study, we firstly analyzed the characteristics and survival of NSCLC patients with NTRK family mutations. Moreover, the association between NTRK family mutations and ICIs efficacy was investigated using an ICI-treated patient cohort and further validated by another cohort. Mechanically, we explored the difference of PD-L1 expression, tumor mutational burden (TMB), blood TMB (bTMB) and immune-related signatures between NTRK mutated and NTRK wild-type (WT) NSCLC patients.

## Patients and methods

### Data sources

Next-generation sequencing data of 1567 NSCLC patients (**Zehir cohort**) was obtained through cBioPortal for Cancer Genomics (https://www.cbioportal.org/) [24]. One available immunotherapy cohort (**Samstein cohort**) [25] with 350 NSCLC patients who received ICIs treatment at the Memorial Sloan Kettering Cancer Center (MSKCC) and another immunotherapy cohort (**OAK/POPLAR cohort**) with 569 NSCLC patients who underwent atezolizumab from OAK and POPLAR [26] were included in this study. Detailed information for each cohort was shown in Table 1. Whole-exome sequencing (WES) data and RNA-seq data of lung adenocarcinoma (LUAD) and lung squamous cell carcinoma (LUSC) were downloaded from The Cancer Genome Atlas (TCGA) (https://portal.gdc.cancer.gov/).

**Table 1:** The detailed information of each cohort in this study

Characteristic	Zehir Cohort N = 1567	Samstein Cohort N = 350	OAK/POPLAR cohort N = 569
**Age (median)**		67 (range, 31–90)	63 (range, 33–82)
< 65	NA	143 (40.9%)	322 (56.6%)
≥ 65	NA	207 (59.1%)	247 (43.4%)
**Gender**			
Female	886 (56.5%)	180 (51.4%)	215 (37.8%)
Male	681(43.5%)	170 (48.6%)	354 (62.2%)
**Smoking status**			
Prev/Current	972 (62.0%)	NA	458 (80.5%)
Never	334 (21.3%)	NA	111 (19.5%)
Unknown	261 (16.7%)		
**Histology**			
LUAD	1268 (80.9%)	271 (77.4%)	
Non-squamous			408 (71.7%)
LUSC	123 (8.1%)	45 (12.9%)	161 (28.3%)
Others	176 (11.2%)	34 (9.7%)	
**Treatment type**			
PD1/PD-L1	NA	329 (94.0%)	569 (100.0%)
Combination	NA	21 (6.0%)	
**PD-L1 expression**			
TC0 and IC0	NA	NA	180 (31.6%)
TC1/2/3or IC1/2/3	NA	NA	241 (42.4%)
Unknown			4 (0.7%)
**TMB**	1567 (100.0%)	350 (100%)	NA
**bTMB**	NA	NA	429 (75.4%)
**Gene mutation**			
NTRK1 MT	55 (3.5%)	13 (3.7%)	8 (1.4%)
NTRK2 MT	23 (1.5%)	9 (2.6%)	14 (2.5%)
NTRK3 MT	79 (5.0%)	20 (5.7%)	40 (7.0%)
NTRK MT	148 (9.4%)	38 (10.9%)	58 (10.2%)
NTRK WT	1419 (90.6%)	312 (89.1%)	511 (89.8%)
**Overall Survival**	1443 (92.1%)	350 (100.0%)	569 (100.0%)

NA: not available; MT: mutation; WT: wild-type; LUAD: lung adenocarcinoma; LUSC: Lung squamous cell carcinoma

### PD-L1 expression analysis

The detection method of PD-L1 was described in the previously published literature [27]. Specifically, the PD-L1 expression was detected by Ventana SP142 PD-L1 immunohistochemistry (IHC). The PD-L1 expression in tumor cells (TC) or tumor-infiltrating cells (IC) was classified by an IHC-scoring convention. PD-L1 scoring for both TC and IC was as follows: TC0/IC0 was defined as < 1% PD-L1 expression on TC or IC. TC1/2 or IC1/2 was defined as ≥ 1% and < 50% PD-L1 expression on TC or IC; TC3/IC3 was defined as ≥ 50% PD-L1 expression on TC or IC.

### Gene mutation and TMB analyses

The gene alteration frequency and co-occurrence genes were analyzed by cBioportal database. Mutation profiles were assessed by next-generation sequencing in Zehir cohort and by WES in TCGA cohort. TMB was defined as total number of nonsynonymous somatic, somatic gene coding errors, base substitution, gene insertion or deletion errors detected per million bases [28]. Furthermore, as previous literature described, the data of bTMB from OAK/POPLAR cohort between patients with NTRK MT and WT was compared [26].

### Immune-related signature analysis

To investigate the association between NTRK family mutations and immune-related signatures, we evaluated tumor infiltrating leukocytes and immune related genes using the TCGA cohort. The differences in composition of immune cell infiltrates between NTRK MT and WT were analyzed using the analytical CIBERSORT platform (https://cibersort.stanford.edu/) [29]. TIMER database was further used to validate the correlation between NTRK MT and immune infiltrates [30]. Furthermore, as reported in previous studies [31], the association between cytolytic activity (GZMA, PRF1), immune checkpoint (CD274, CTLA4, IDO1, PDCD1LG2, LAG3, PDCD1, HAVCR2 and TIGIT), chemokine (CXCL9, CXCL10 and CCR5) and NTRK MT was analyzed in NSCLC patients from the TCGA cohort.

### Statistical analysis

The chi-squared test was used to perform the association between clinical variables and NTRK MT. The nonparametric test was used to analyze TMB, bTMB difference between NTRK MT and WT. The Wilcoxon test was used to conduct statistical analysis of comparisons between two groups. The Kaplan-Meier curves and log-rank test were conducted to perform survival analysis. Pearson chi-square analysis was performed to evaluate the difference in PD-L1 expression between NTRK MT and NTRK WT. The R version 4.1.2 and Graphpad prism 8.0 were used to perform all analyses. A *P-*value < 0.05 was considered statistically significant.

## Results

### NTRK mutation frequencies and correlation with clinical variables and prognosis of patients with NSCLC

**Fig. 1:**
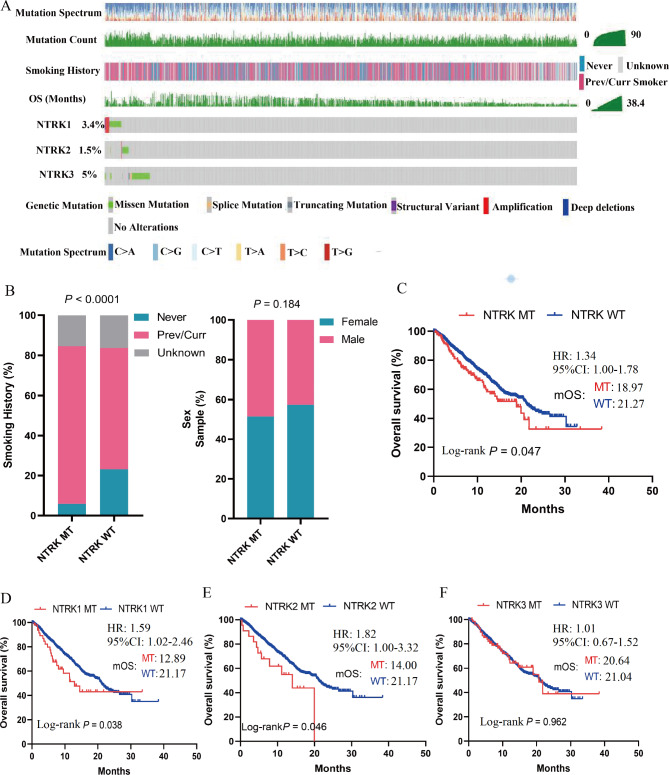
The characteristics of NTRK mutation in patients with non-small cell lung cancer (NSCLC). **A**: The mutation spectrum of NTRK mutation in NSCLC patients. **B**: The association between NTRK mutation and smoking history, sex. **C**: Survival analysis in NTRK altered and unaltered patients with NSCLC. **D-F**: Survival analysis in NTRK1/2/3 MT and WT patients with NSCLC.

The prevalence of NTRK family mutations was evaluated in patients with NSCLC from Zehir cohort. Of all 1567 patients, there were 55 cases (3.5%) with NTRK1 mutation, 23 cases (1.5%) with NTRK2 mutation, and 79 cases (5.0%) with NTRK3 mutation (Table 1). As shown in Fig. 1A, the waterfall depicted the association of the NTRK mutation spectrum in NSCLC with multiple variables, including smoking history, gender and survival. In detail, more previous/current smokers were found in the NTRK MT group compared with the NTRK WT group (*P* < 0.001) while there is no difference in NTRK mutation frequency between males and females (Fig. 1B). Furthermore, survival analysis was performed utilizing 1443 individuals with precise survival information from this cohort based on their NTRK mutation status. Surgery, chemotherapy, targeted therapy, and/or immunotherapy were the principal treatments for these individuals. Notably, the survival of patients with NTRK MT was worse to that of WT patients (mOS, MT vs. WT: 18.97 vs. 21.27 months, HR = 1.34, 95%CI 1.00-1.78; log-rank *P* = 0.047) (Fig. 1C). Furthermore, the relationship between NTRK MT subtypes and survival was analyzed. The results showed compared with WT patients, patients with NTRK1 MT (mOS, MT vs. WT: 12.89 vs. 21.17 months, HR = 1.59, 95%CI 1.02–2.46; log-rank *P* = 0.038) or NTRK2 MT (mOS, MT vs. WT: 14.00 vs. 21.17 months, HR = 1.82, 95%CI 1.00-3.32; log-rank *P* = 0.046) had a significantly worse OS (Fig. 1D-E). However, no significant difference in survival was observed between patients with NTRK3 MT and NTRK3 WT patients (mOS, MT vs. WT: 20.64 vs. 21.04 months, HR = 1.01, 95%CI 0.67–1.52; log-rank *P* = 0.962) (Fig. 1F). These findings suggested that patients with NTRK family mutations had worse prognosis compared to WT patients if not defining specific treatments.

### Impact of NTRK mutations on outcomes of patients treated with ICIs in Samstein cohort

The Samstein cohort, involving 350 advanced NSCLC patients treated with ICIs, was used to investigate the impact of NTRK MT on ICIs efficacy. The detailed baseline characteristics were described in Table 1. In brief, this cohort was representative of advanced NSCLC patients with median age of 67 years (range, 31–90) and major proportion of LUAD (77.4%), in which 94.0% of patients received anti-PD-1/PD-L1 monotherapy and the rest underwent anti-PD-1/PD-L1 and anti-CTLA-4 combination therapy. Among them, there were 13 cases with NTRK1 mutation (3.7%), 9 cases with NTRK2 mutation (2.6%), and 20 cases with NTRK3 mutation (5.7%).

**Fig. 2:**
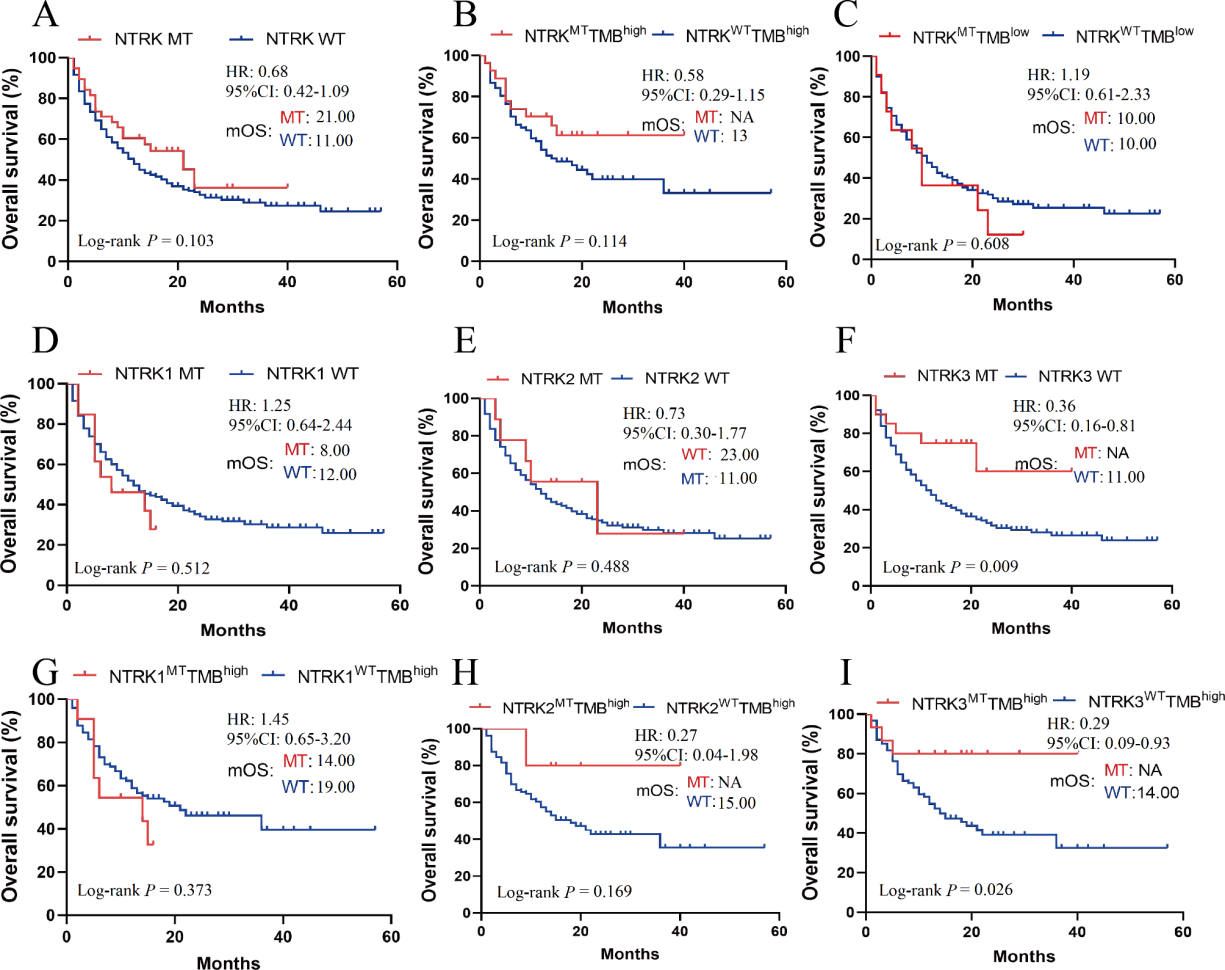
Survival analysis for NTRK mutant patients received ICIs in Samstein cohort. **A**: Survival curves for patients with or without NTRK mutation receiving ICIs treatment. **B**: Survival curves of NSCLC patients in the TMB-high group. **C**: Survival curves of NSCLC patients in the TMB-low group. **D-F**: Survival curves for patients with or without NTRK1/2/3 mutation receiving ICIs treatment. **G-I**: Survival curves of subgroup analysis in NSCLC patients with high-TMB.

As shown in Fig. 2A, the median OS for the 38 NTRK MT patients receiving ICIs was 21 months, whereas it was only 11 months for the 312 WT patients treated with ICIs. However, the difference in OS between NTRK MT and WT patients was not statistically significant (HR = 0.68, 95%CI 0.42–1.09; log-rank *P* = 0.103). TMB is strongly associated with immunogenicity and currently confirmed as a powerful biomarker for ICIs efficacy. However, TMB (cutoff = 10) did not identify the ICIs beneficiaries of NSCLC patients with NTRK family mutations (high-TMB group mOS, MT vs. WT: NA vs. 13.00 months, HR = 0.58, 95%CI 0.29–1.15; log-rank *P* = 0.114; low-TMB group mOS, MT vs. WT: 10.00 vs. 10.00 months, HR = 1.19, 95%CI 0.61–2.33; log-rank *P* = 0.608) in this cohort (Fig. 2B-C).

Next, subgroup survival analysis was performed to explore the impact of NTRK mutation subtype on the efficacy of ICIs. In both NTRK1 subgroup (mOS, MT vs. WT: 8.00 vs. 12.00 months, HR = 1.25, 95%CI 0.64–2.44; log-rank *P* = 0.512) (Fig. 2D) and NTRK2 subgroup (mOS, MT vs. WT: 23.00 vs. 11.00 months, HR = 0.73, 95%CI 0.30–1.77; log-rank *P* = 0.488) (Fig. 2E), there was no significant difference in survival between MT and WT. However, ICIs significantly prolonged OS in patients with NTRK3 MT compared to patients with WT (mOS: not available vs. 11.00 months, HR = 0.36, 95%CI 0.16–0.81; log-rank *P* = 0.009) (Fig. 2F), indicating patients with NTRK3 could benefit more from ICIs treatment.

Furthermore, in TMB-high subgroup (> 10) analysis, no significant difference in survival was observed between patients with NTRK1 MT and WT (mOS: 14.00 vs. 19.00 months, log-rank *P* = 0.373) (Fig. 2G), or between patients with NTRK2 MT and WT (mOS: NA vs. 15.00 months, log-rank *P* = 0.169) (Fig. 2H). Notably, the mOS of patients with NTRK3 MT and high TMB was significantly prolonged compared to that of WT patients (mOS: NA vs. 14.00 months, HR = 0.29, 95%CI 0.09–0.93; log-rank *P* = 0.026) (Fig. 2I). However, in low-TMB subgroup (< 10), there was no significant difference in survival between NTRK1/2/3 MT and NTRK1/2/3 WT patients (Supplementary Fig. 1A-C). These results suggested that among patients subject to ICIs treatment in Samstein cohort, NTRK mutant patients’ survival was comparable to or superior to that of WT patients, particularly those with NTRK3 MT.

### Impact of NTRK mutations on outcomes of patients treated with ICIs in OAK/POPLAR cohort

**Fig. 3:**
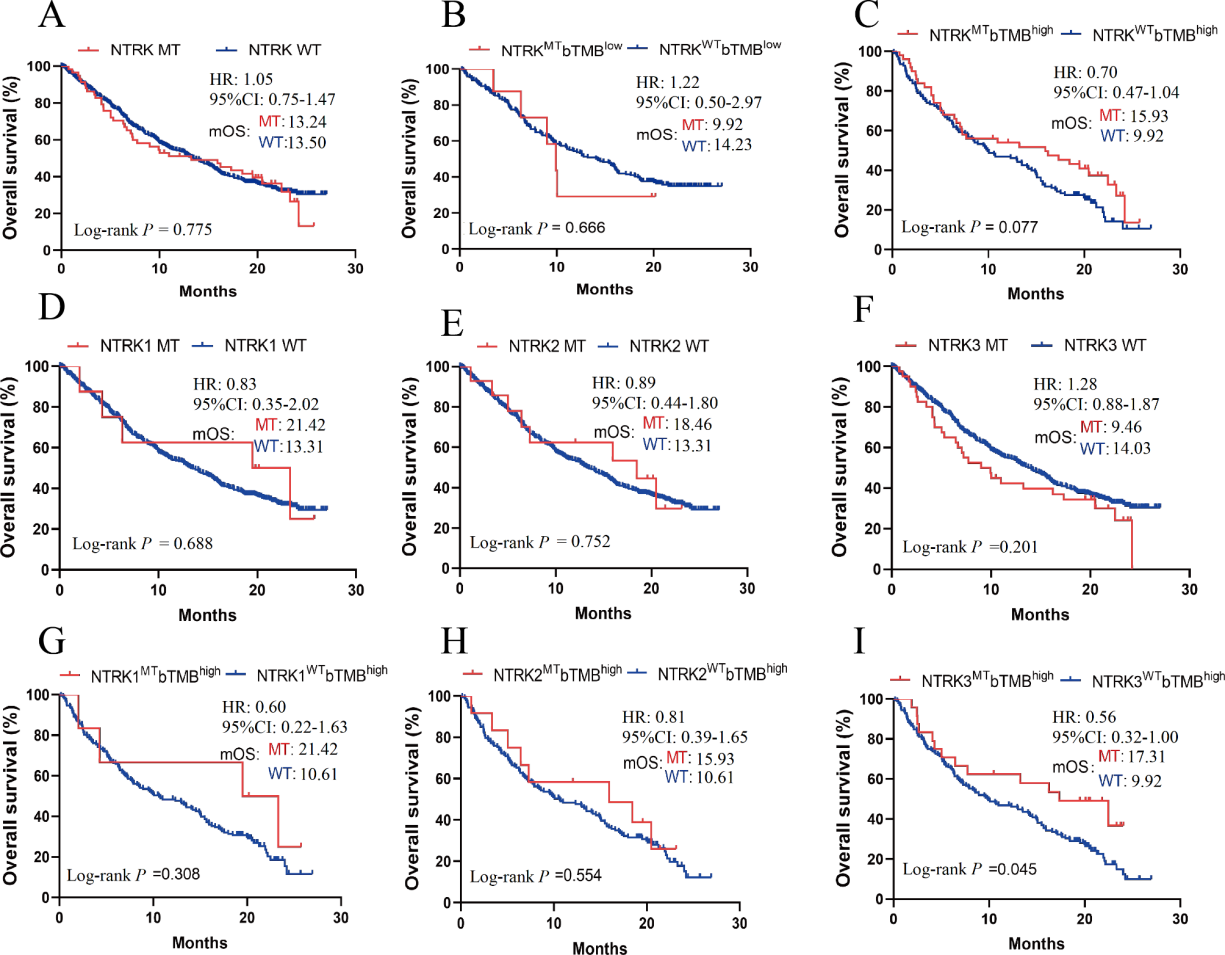
Survival analysis for NTRK mutant patients received ICIs in OAK/ POPLAR cohorts. **A**: Survival curves for patients with or without NTRK mutation receiving ICIs treatment. **B**: Survival curves of NSCLC patients in the bTMB-low group. **C**: Survival curves of NSCLC patients in the bTMB-high group. **D-F**: Survival curves for patients with or without NTRK1/2/3 mutation receiving ICIs treatment. **G-I**: Survival curves of subgroup analysis in NSCLC patients with high-bTMB.

To further validate the impact of NTRK family mutations on ICIs efficacy, 569 NSCLC patients who received atezolizumab from OAK/POPLAR cohort were analyzed. In this cohort, the mutation rates of NTRK were 1.4% (NTRK1), 2.5% (NTRK2) and 7.0% (NTRK3), and the detailed characteristics of patients were shown in Table 1. As shown in Fig. 3A, the survival curves for ICIs-treated NSCLC patients with NTRK MT and WT were very identical and statistical analysis showed there was no difference between their mOS (mOS, MT vs. WT: 13.24 vs. 13.50 months, HR = 1.05, 95%CI 0.75–1.47; log-rank *P* = 0.775). When patients were stratified according to bTMB (cutoff = 10), there was also no significant difference in survival between individuals with NTRK MT and NTRK WT, whether in the low or high subgroup (low bTMB group, mOS, MT vs. WT: 9.92 vs. 14.23 months, *P* = 0.666; high-bTMB group, mOS, MT vs. WT: 15.93 vs. 9.92 months, log-rank *P* = 0.077) (Fig. 3B-C), which is consistent with the results of Samstein cohort.

Next, subgroup survival analysis was performed based on NTRK mutation subtypes. Compared to WT patients, there was a tendency of prolonged mOS but not statistically significant in NTRK1 MT patients (mOS, MT vs. WT: 21.42 vs. 13.31 months, log-rank *P* = 0.688) and NTRK2 MT patients (mOS, MT vs. WT: 18.46 vs. 13.31 months, log-rank *P* = 0.752) (Fig. 3E-D), whereas the trend of OS benefit was not observed in patients with NTRK3 MT (mOS, MT vs. WT: 9.46 vs. 14.03 months, log-rank *P* = 0.201) (Fig. 3F). Additionally, the progression-free survival (PFS) was also analyzed and the results showed that the mPFS was comparable between NTRK MT and NTRK WT patients (all *P* > 0.05) (Supplementary Fig. 2A-C).

For further exploration, we conducted subgroup analyses in patients with high bTMB and low bTMB (the cut-off = 10). In patients with high bTMB, no statistical difference was observed in patients with NTRK1 MT (mOS, MT vs. WT: 21.42 vs. 10.61 months, log-rank *P* = 0.308) (Fig. 3G) and with NTRK2 MT (mOS, MT vs. WT: 15.93 vs. 10.61 months, log-rank *P* = 0.554) (Fig. 3H). Importantly, in high bTMB subgroup, significant OS prolongation was found in patients with NTRK3 MT compared to those with NTRK3 WT (mOS: 17.31 vs. 9.92 months, HR = 0.56, 95%CI 0.32-1.00; log-rank *P* = 0.045) (Fig. 3I). However, in patients with low bTMB, no significant difference was found between NTRK1/2/3 MT and WT subgroups, which might be due to small size samples (Supplementary Fig. 3A-C). Moreover, we conducted subgroup analyses based on PD-L1 expression. However, no significant difference in survival was observed between NTRK family mutation and WT, regardless of with high PD-L1 expression or low expression (all *P* < 0.05) (Supplementary Fig. 4A-H). Taken together, the above findings indicated that patients with NTRK MT could benefit similarly or even more from ICIs treatment compared to patients with NTRK WT, while the efficacy of atezolizumab was not obvious in NTRK3 MT patients, which differed from the findings in Samstein cohort.

### PD-L1 expression, TMB and bTMB across NTRK mutation subtypes from different cohorts

**Fig. 4:**
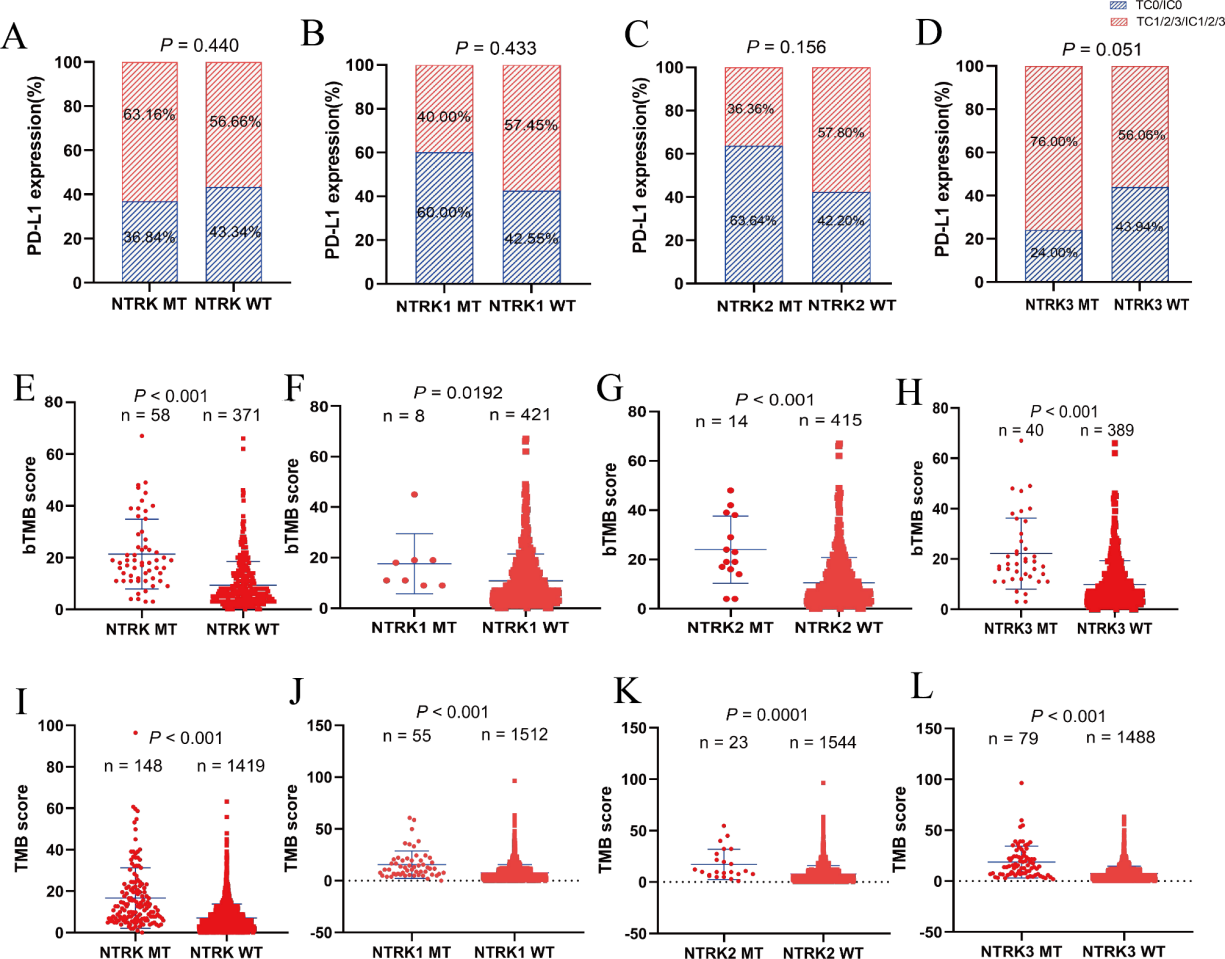
The association between NTRK mutation and PD-L1 expression, tumor mutational burden (TMB), blood TMB (bTMB). **A-D**: The association between NTRK, NTRK1, NTRK2, NTRK3 mutation and PD-L1 expression. **E-H**: The association between NTRK, NTRK1, NTRK2, NTRK3 mutation and bTMB levels (all *P* < 0.05). **I-L**: The association between NTRK, NTRK1, NTRK2, NTRK3 mutation and TMB levels (all *P* < 0.001)

Using the data from the OAK cohort, the expression of PD-L1 was analyzed. No significant differences in the PD-L1 expression were observed between NTRK MT, NTRK1/2 MT patients and their WT counterparts (Fig. 4A-C). Intriguingly, patients with NTRK3 MT displayed higher level of PD-L1 expression (TC1/2/3 or IC1/2/3: 76.00%) than those those with NTRK3 WT (TC1/2/3 or IC1/2/3: 56.06%) (Fig. 4D).

Next, the association of NTRK mutation with bTMB, another important biomarker predicting immunotherapy efficacy, was analyzed using OAK/POPLAR cohort. It was revealed that NTRK MT significantly correlated with higher bTMB (*P* < 0.001) (Fig. 4E). Similar results were observed in the subgroup analysis, with significantly higher bTMB in NTRK1 MT, NTRK2 MT, and NTRK3 MT patients than in NTRK1 WT, NTRK2 WT, and NTRK3 WT patients, respectively (all *P* < 0.05) (Fig. 4F-H). In line with above the finding, a significantly higher TMB was observed in patients with NTRK MT compared with those with NTRK WT (*P* < 0.001) (Fig. 4I). Meanwhile, patients in subgroups of NTRK1 MT, NTRK2MT, and NTRK3 MT also had significantly higher TMB compared to their WT counterparts (all *P* < 0.001) (Fig. 4J-L). Similar finding was obtained in the TCGA cohort (all *P* < 0.01) (Supplementary Fig. 5). Moreover, in Samstein cohort, higher TMB was also found in NTRK1 (median TMB: 15.66), NTRK2 (median TMB: 12.97) and NTRK3 MT (median TMB: 19.57). These findings demonstrated that similar PD-L1 expression but higher TMB and bTMB was observed in NTRK mutant NSCLC.

### Immune-related signature of NTRK mutation in TCGA cohort

**Fig. 5:**
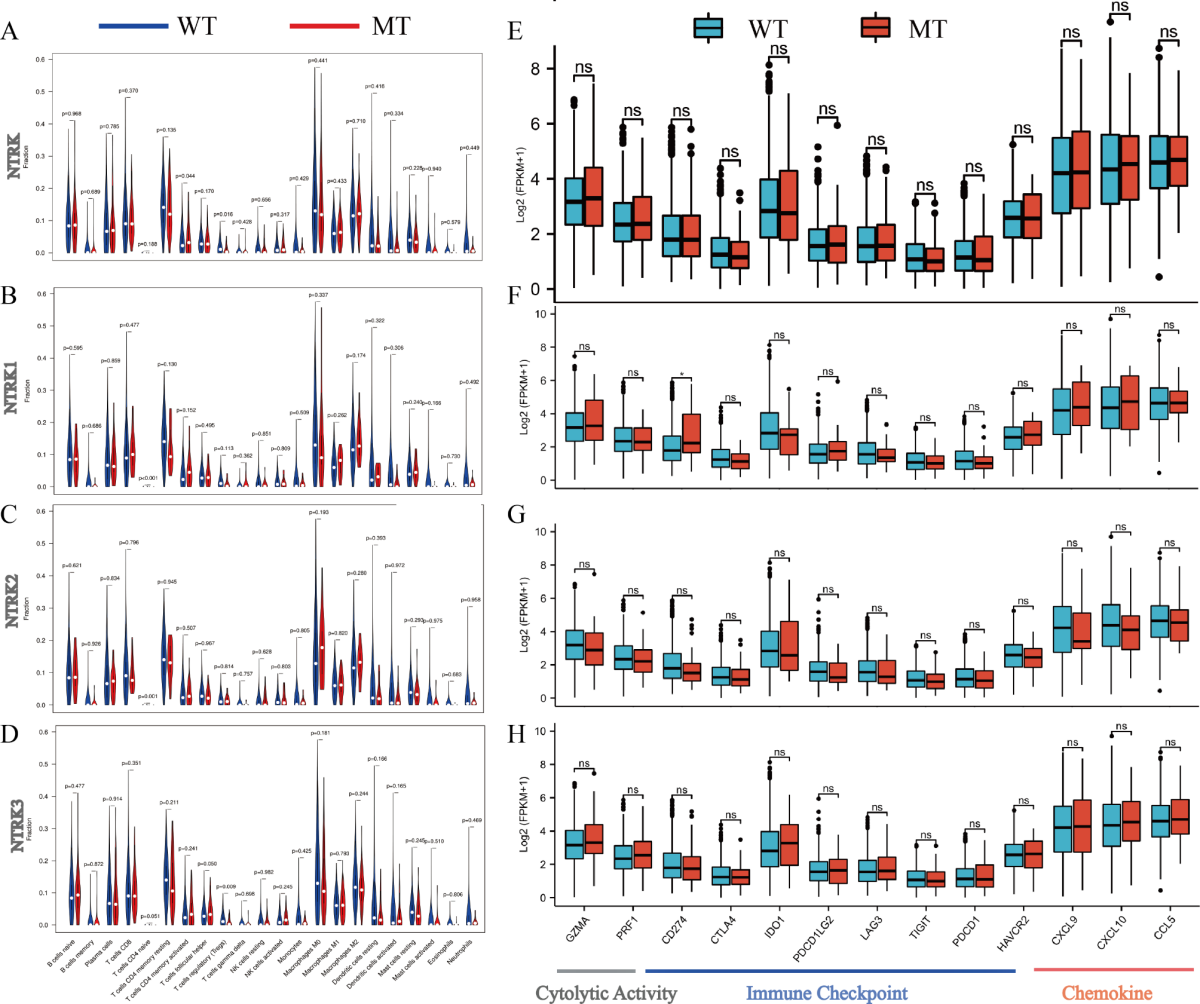
Immune-related signature in the NTRK mutation and wild type of the NSCLC patients from TCGA. **A-C**: Infiltration frequencies of 22 types of immune cells in the NTRK, NTRK1, NTRK2, NTRK3 mutation and wild type. **D-G**: The expression of cytolytic activity related genes, immune checkpoint related genes and chemokine related genes in the NTRK, NTRK1, NTRK2, NTRK3 mutation and wild type of NSCLC patients from TCGA

It is well acknowledged that immune cells play a critical role in regulating the response to immunotherapy. Therefore, CIBERSORT was used to explore the relationship between NTRK MT and of the level of infiltrating immune cells. Notably, a significantly higher infiltration of CD4^+^ memory-activated T cells (*P* = 0.044) and lower regulatory T cell (Tregs) (*P* = 0.016) were observed in NTRK MT NSCLC (Fig. 5A), while the rest of infiltrating immune cells were almost equivalent between NTRK MT and NTRK WT NSCLC (Fig. 5A). Similar analyses were further performed based on NTRK mutation subtypes. The result showed that naïve CD4 T cells (*P* < 0.01) were more abundant in NTRK1 and NTRK2 MT NSCLC, while the infiltration levels of other immune cells in NTRK1 MT or NTRK2 MT NSCLC were comparable with corresponding WT NSCLC (all *P* > 0.05) (Fig. 5B-C). Moreover, there was an evident trend that naïve CD4 T cells (*P* = 0.051) and follicular helper T cells (*P* = 0.050) were more abundant in NTRK3 MT NSCLC, while the infiltration of Tregs (*P* = 0.009) was more enriched in NTRK3 WT NSCLC (Fig. 5D). Furthermore, TIMER database was used to validate the relationship between NTRK3 MT and the infiltration of immune cells. The results confirmed that NTRK3 MT was positively related to naïve CD8 T cells (*P* = 0.02) and negatively related to the infiltration of Tregs (*P* = 0.00034) (Supplementary Fig. 6A and B).

The TCGA cohort was used to further investigate the differences in immune-related signature between patients with NTRK MT and WT NSCLC. The immune-related signatures exhibited similar levels (namely, immune-checkpoint signatures) or displayed a higher inclination (GZMA, PRF1, CXCL9, CXCL10, and CCL5) in NTRK MT NSCLC in comparison to NTRK WT NSCLC. However, none of the aforementioned results achieved statistical significance (Fig. 5E). Likewise, subgroup analysis was performed based on NTRK MT subtypes. Similar findings were obtained, with most immune-related signatures in NTRK1 MT NSCLC being equivalent (CD274, CTLA4, IDO1, LAG3, TIGIT, and PDCD1) or even more abundant (GZMA, PRF1, CXCL9, and CXCL10) compared with those in NTRK1 WT NSCLC (Fig. 5F). Regarding NTRK2, most immune checkpoint signatures (CD274, CTLA4, IDO1, PDCD1LG2, LAG3, TIGIT, PDCD1, and HAVCR2) were mildly lower in NTRK MT NSCLC than in NTRK WT NSCLC (Fig. 5G). In terms of NTRK3, the cytolytic activity related genes (GZMA, PRF1) and chemokine related genes (CXCL9, CXCL10) were more enriched in NTRK3 MT NSCLC (Fig. 5H). Collectively, the anti-tumor immunity in NTRK1/2/3 MT NSCLC was enhanced in some aspect compared with their WT counterparts, especially in patients with NTRK3 MT.

## Discussion

Oncogenic NTRK gene mutations have been identified in multiple types of cancers, including NSCLC [18, 32]. Though larotrectinib and entrectinib have shown considerable efficacy in patients harboring NTRK fusions, acquired resistance to TRK inhibitors is inevitable [17, 23]. Moreover, not all NTRK family mutations are responsive to targeted therapy. A number of studies have demonstrated a negative correlation between specific driver mutations and ICIs efficacies [6, 33]. However, there is limited evidence whether patients with NTRK family mutations could benefit from ICI treatment. To the best of our knowledge, this was the first study to comprehensively investigate the correlation between NTRK family mutations and ICIs efficacy and explore its possible mechanisms using different cohorts. NSCLC patients with mutations of NTRK family and its three subtypes, NTRK1, NTRK2 and NTRK3, were analyzed, and it was discovered that the overall NTRK mutations or three subtypes were associated with comparable or longer patient OS with ICIs treatment compared to WT.

In a single-institution retrospective study by Dudnik. et al., the objective response rate was 50% in two NTRK mutant NSCLC patients treated with ICIs, and the median PFS and OS were not yet reached [34]. This finding implied that ICIs could be beneficial in NTRK-mutant patients. In this study, our results demonstrated that the survival of NTRK mutant NSCLC patients treated with ICIs in Samstein cohort was comparable to or superior to that of WT patients, particularly those with NTRK3 MT. A previous study exploring the relationship between driver mutation and ICIs efficacy in LUAD also revealed a strong association between NTRK3 mutation and ICIs benefit, which was consistent with our subgroup analysis results [35]. The results from OAK/POPLAR cohort further validated the relationship between NTRK MT and ICI efficacy, and the overall results were similar to those of the Samstein cohort. These findings prove that NTRK MT has no significant impact on the efficacy of ICIs; while other mutations, such as EGFR and ALK, have shown a considerable detrimental effect on ICIs benefit and might even result in hyperprogressive disease [36, 37]. The discrepancy in the impact of NTRK3 MT on ICIs efficacy across two immunotherapy cohorts might be due to the differences in patient characteristics, especially the expressions of important ICIs biomarkers, such as TMB and PD-L1 expression.

A number of clinical studies have revealed that patients with high TMB have greater response rate and better survival following ICI treatment [38–40]. TMB, rather than PD-L1 expression, was proven to be a more effective biomarker for predicting response to ICIs in EGFR mutant NSCLC [41]. Therefore, we investigated the efficacy of ICIs in NTRK mutant patients with high TMB or bTMB level (> 10). In NTRK1/2 mutant NSCLC with high TMB or bTMB, a similar or longer median OS was observed compared with NTRK1/2 WT. Remarkably, in TMB or bTMB high subgroup, the median OS of patients with NTRK3 MT was superior to that of WT patients, which might be due to that NTRK3 mutant patients had higher TMB or bTMB than patients with NTRK1/2 mutations.

Indeed, in our study, we found that PD-L1 expression was not closely related to the ICIs efficacy in patients with NTRK MT. Likewise, a prior study reported a lack of response in PD-L1 positive or high, EGFR mutant patients administered with pembrolizumab [10]. Moreover, in ALK-positive NSCLC, elevated PD-L1 expression did not correlate with improved clinical benefit with ICIs [42, 43]. These results indicated that PD-L1 expression might not be a suitable biomarker for ICIs benefit in NSCLC with driver mutations. Additionally, other crucial factors also affect ICIs efficacy, most notably the key compositions of the tumor microenvironment (TME) [44].

The TME influence response and resistance to ICIs by its various components, including T cells, myeloid lineage cells, and cytokines and chemokines [45]. Notably, it has been shown that the majority of cancers harboring driver mutations are associated with an immunosuppressive microenvironment [11]. For example, tumor-infiltrating lymphocytes were dramatically reduced in EGFR-mutated lung cancer [12]; whereas CD8 + T cells were either absent or functionally impaired in ALK-positive lung cancer [11, 46]. In our analysis, the infiltration level of anti-tumor immune cells, particularly CD8 + T cells, was not significantly reduced in NTRK mutant patients. Memory T cells can optimize strategies for immunotherapy against cancer. CD4 memory activated T cells have been reported to increase in number and differentiate into certain CD4 + T cell subpopulations targeting specific pathogens after secondary antigen stimulation [47]. Deep analysis of the activation and regulation of memory T cells has the potential to significantly improve infection vaccines, cancer immunotherapies, and autoimmune therapies [48]. By inhibiting antitumor immunity, Tregs are widely known to play a crucial role in tumor progression [49]. Our findings revealed that NTRK MT patients had a significantly higher infiltration of CD4^+^ memory-activated T cells and lower infiltration of Tregs. Additionally, patients with NTRK1/2 had higher infiltration of naïve CD4 + T cells compared with their WT counterparts. After antigen stimulation, naïve CD4 + T cells proliferate and differentiate into a variety of subpopulations that play vital roles in adaptive immunity. Moreover, patients with NTRK3 MT showed an increased follicular helper T cell and decreased Tregs infiltration. The abundance of follicular helper T cells has been reported to be associated with better prognosis of lung cancer [50]. In addition, NTRK mutant NSCLC patients exhibited increased expression of cytolytic activity markers (GZMA, PRF1), immunological checkpoints (CD274), and chemokines (CXCL9, CXCL10). Taken together, these findings may explain in part why patients with NTRK mutation, especially NTRK3, would benefit more from ICIs treatment. Further mechanical researches involving NTRK family mutation and TME are required to elucidate how NTRK modulates response to ICIs.

The present study had some limitations. Firstly, the methodology and retrospective setting of this study might introduce multiple biases. Secondly, due to the low frequency of NTRK mutations, fewer NTRK mutant patients were treated with ICIs, which may also lead to bias. Thus, further research with larger sample size is needed to validate these findings. Thirdly, due to the limitation of data sources, the exact gene fusion information for NTRK could not be obtained, which was not completely consistent with the NTRK fusion-positive patients treated with targeted TKIs in clinical practice. However, our results of NTRK mutant populations included NTRK fusion-positive patients. Therefore, our study would offer insight on whether patients with NTRK mutations can benefit from ICIs and implies that they get it on a par with or more than WT patients. Additionally, to conduct further in vitro and in vivo studies to validate the role of NTRK mutations in ICI effectiveness is necessary in the future.

## Conclusions

In summary, we comprehensively investigate the efficacy of ICIs treatment in NSCLC patients harboring NTRK MT across two immunotherapy cohorts. Our study was the one of first reports that provided evidence for the application of ICIs in NSCLC patients with NTRK family mutations. According to our analyses, NSCLC patients with NTRK MT demonstrated a comparatively extended OS compared to those with NTRK WT, especially when taking high TMB or bTMB into consideration. These findings may provide clinicians with hints that NTRK MT patients could potentially derive similar benefits from ICIs as WT individuals, in contrast to patients with other gene mutations, such as EGFR mutation.

### Electronic supplementary material

Below is the link to the electronic supplementary material.

**Supplementary Material 1**: Survival curves of NTRK1/2/3 mutated NSCLC patients in the TMB-low group

**Supplementary Material 2**: The progression-free survival (PFS) comparison between patients with or without NTRK1/2/3 mutation who receiving atezolizumab

**Supplementary Material 3**: Survival curves of NTRK1/2/3 mutated NSCLC patients in the bTMB-low group

**Supplementary Material 4**: Survival curves of NTRK1/2/3 mutated NSCLC patients based on PD-L1 expression

**Supplementary Material 5**: The association between TMB and NTRK mutation in NSCLC patients from TCGA cohort

**Supplementary Material 6**: The validation of relationship between NTRK3 mutation and infiltration of immune cells from TIMER database

## Data Availability

The study was conducted based on publicly available datasets, including cBioportal (https://www.cbioportal.org/), TCGA (https://portal.gdc.cancer.gov/) and TIMER (https://timer.cistrome.org/). The detailed datasets that supporting this study were described in the method section.

## References

[1] Sung H, Ferlay J, Siegel RL, Laversanne M, Soerjomataram I, Jemal A (2021). : Global Cancer statistics 2020: GLOBOCAN estimates of incidence and Mortality Worldwide for 36 cancers in 185 countries. CA Cancer J Clin.

[2] Chen Z, Fillmore CM, Hammerman PS, Kim CF, Wong KK (2014). Non-small-cell Lung Cancers: a heterogeneous set of Diseases. Nat Rev Cancer.

[3] Miller M, Hanna N (2021). Advances in systemic therapy for non-small cell Lung cancer. BMJ.

[4] Rangachari D, Costa DB (2019). From Hope to reality: durable overall survival with Immune Checkpoint inhibitors for Advanced Lung Cancer. J Clin Oncol.

[5] Grant MJ, Herbst RS, Goldberg SB (2021). Selecting the optimal immunotherapy regimen in driver-negative metastatic NSCLC. Nat Rev Clin Oncol.

[6] Seegobin K, Majeed U, Wiest N, Manochakian R, Lou Y, Zhao Y (2021). Immunotherapy in Non-small Cell Lung Cancer with actionable mutations other Than EGFR. Front Oncol.

[7] Dantoing E, Piton N, Salaun M, Thiberville L, Guisier F. Anti-PD1/PD-L1 immunotherapy for Non-small Cell Lung Cancer with Actionable Oncogenic driver mutations. Int J Mol Sci 2021, 22(12). 10.3390/ijms22126288 PMC8230861 34208111

[8] Lee CK, Man J, Lord S, Links M, Gebski V, Mok T (2017). Checkpoint inhibitors in metastatic EGFR-Mutated Non-small Cell Lung Cancer-A Meta-Analysis. J Thorac Oncol.

[9] Lee CK, Man J, Lord S, Cooper W, Links M, Gebski V (2018). Clinical and molecular characteristics Associated with Survival among patients treated with checkpoint inhibitors for Advanced Non-small Cell Lung Carcinoma: a systematic review and Meta-analysis. JAMA Oncol.

[10] Lisberg A, Cummings A, Goldman JW, Bornazyan K, Reese N, Wang T (2018). A phase II study of Pembrolizumab in EGFR-Mutant, PD-L1+, tyrosine kinase inhibitor naive patients with Advanced NSCLC. J Thorac Oncol.

[11] Liu SY, Dong ZY, Wu SP, Xie Z, Yan LX, Li YF (2018). Clinical relevance of PD-L1 expression and CD8 + T cells infiltration in patients with EGFR-mutated and ALK-rearranged Lung cancer. Lung Cancer.

[12] Dong ZY, Zhang JT, Liu SY, Su J, Zhang C, Xie Z (2017). : EGFR mutation correlates with uninflamed phenotype and weak immunogenicity, causing impaired response to PD-1 blockade in non-small cell Lung cancer. Oncoimmunology.

[13] Mazieres J, Drilon A, Lusque A, Mhanna L, Cortot AB, Mezquita L (2019). Immune checkpoint inhibitors for patients with advanced Lung cancer and oncogenic driver alterations: results from the IMMUNOTARGET registry. Ann Oncol.

[14] Yamada T, Hirai S, Katayama Y, Yoshimura A, Shiotsu S, Watanabe S (2019). Retrospective efficacy analysis of immune checkpoint inhibitors in patients with EGFR-mutated non-small cell Lung cancer. Cancer Med.

[15] Dudnik E, Peled N, Nechushtan H, Wollner M, Onn A, Agbarya A (2018). : BRAF Mutant Lung Cancer: programmed death Ligand 1 expression, Tumor Mutational Burden, microsatellite instability status, and response to Immune check-point inhibitors. J Thorac Oncol.

[16] Zhang C, Wang H (2021). Regulation of immune microenvironment may enable MET-altered NSCLC patients to benefit from immune checkpoint inhibitors. Lung Cancer.

[17] Cocco E, Scaltriti M, Drilon A (2018). NTRK fusion-positive cancers and TRK inhibitor therapy. Nat Rev Clin Oncol.

[18] Solomon JP, Benayed R, Hechtman JF, Ladanyi M (2019). Identifying patients with NTRK fusion cancer. Ann Oncol.

[19] Frampton JE (2021). Entrectinib: a review in NTRK + solid tumours and ROS1 + NSCLC. Drugs.

[20] Drilon A, Laetsch TW, Kummar S, DuBois SG, Lassen UN, Demetri GD (2018). Efficacy of Larotrectinib in TRK Fusion-positive cancers in adults and children. N Engl J Med.

[21] Rolfo CD, Doebele FGDBRC, Drilon AE, Siena S, Patel M, Cho BC et al. Efficacy and safety of entrectinib in patients (pts) with NTRK-fusion positive (NTRK-fp) solid tumors: an updated integrated analysis. J Clin Oncol 2020, 38((15_suppl)).

[22] Drilon A, Albert SKCM, Nagasubramanian R, Hechtman JF, Reeves JA, Beckmann G, Rudolph M et al. Abstract CT020: long-term outcomes of patients with TRK fusion cancer treated with larotrectinib. Cancer Res 2021, 81(13 Supplement, CT020-CT020).

[23] Cocco E, Schram AM, Kulick A, Misale S, Won HH, Yaeger R (2019). : resistance to TRK inhibition mediated by convergent MAPK pathway activation. Nat Med.

[24] Zehir A, Benayed R, Shah RH, Syed A, Middha S, Kim HR (2017). Mutational landscape of metastatic cancer revealed from prospective clinical sequencing of 10,000 patients. Nat Med.

[25] Samstein RM, Lee CH, Shoushtari AN, Hellmann MD, Shen R, Janjigian YY (2019). Tumor mutational load predicts survival after immunotherapy across multiple cancer types. Nat Genet.

[26] Gandara DR, Paul SM, Kowanetz M, Schleifman E, Zou W, Li Y (2018). Blood-based Tumor mutational burden as a predictor of clinical benefit in non-small-cell Lung cancer patients treated with atezolizumab. Nat Med.

[27] Rittmeyer A, Barlesi F, Waterkamp D, Park K, Ciardiello F, von Pawel J (2017). Atezolizumab versus Docetaxel in patients with previously treated non-small-cell Lung cancer (OAK): a phase 3, open-label, multicentre randomised controlled trial. Lancet.

[28] Yarchoan M, Hopkins A, Jaffee EM (2017). Tumor mutational burden and response rate to PD-1 inhibition. N Engl J Med.

[29] Newman AM, Liu CL, Green MR, Gentles AJ, Feng W, Xu Y (2015). Robust enumeration of cell subsets from tissue expression profiles. Nat Methods.

[30] Li T, Fan J, Wang B, Traugh N, Chen Q, Liu JS (2017). : TIMER: a web server for Comprehensive Analysis of Tumor-infiltrating Immune cells. Cancer Res.

[31] Thorsson V, Gibbs DL, Brown SD, Wolf D, Bortone DS, Ou Yang TH (2018). The Immune Landscape of Cancer. Immunity.

[32] Harada G, Santini FC, Wilhelm C, Drilon A (2021). NTRK fusions in Lung cancer: from biology to therapy. Lung Cancer.

[33] Qiao M, Jiang T, Liu X, Mao S, Zhou F, Li X (2021). Immune checkpoint inhibitors in EGFR-Mutated NSCLC: Dusk or Dawn?. J Thorac Oncol.

[34] Dudnik E, Bshara E, Grubstein A, Fridel L, Shochat T, Roisman LC (2018). Rare targetable drivers (RTDs) in non-small cell Lung cancer (NSCLC): outcomes with immune check-point inhibitors (ICPi). Lung Cancer.

[35] Niu Y, Lin A, Luo P, Zhu W, Wei T, Tang R (2020). Prognosis of lung adenocarcinoma patients with NTRK3 mutations to Immune Checkpoint inhibitors. Front Pharmacol.

[36] Kato S, Goodman A, Walavalkar V, Barkauskas DA, Sharabi A, Kurzrock R (2017). Hyperprogressors after Immunotherapy: analysis of genomic alterations Associated with Accelerated Growth Rate. Clin Cancer Res.

[37] Ferrara R, Mezquita L, Texier M, Lahmar J, Audigier-Valette C, Tessonnier L (2018). : Hyperprogressive Disease in patients with Advanced Non-small Cell Lung Cancer treated with PD-1/PD-L1 inhibitors or with single-Agent Chemotherapy. JAMA Oncol.

[38] Rizvi NA, Hellmann MD, Snyder A, Kvistborg P, Makarov V, Havel JJ (2015). Cancer immunology. Mutational landscape determines sensitivity to PD-1 blockade in non-small cell Lung cancer. Science.

[39] Chowell D, Morris LGT, Grigg CM, Weber JK, Samstein RM, Makarov V (2018). Patient HLA class I genotype influences cancer response to checkpoint blockade immunotherapy. Science.

[40] Carbone DP, Reck M, Paz-Ares L, Creelan B, Horn L, Steins M (2017). First-line nivolumab in stage IV or recurrent non-small-cell Lung Cancer. N Engl J Med.

[41] Hastings K, Yu HA, Wei W, Sanchez-Vega F, DeVeaux M, Choi J (2019). EGFR mutation subtypes and response to immune checkpoint blockade treatment in non-small-cell Lung cancer. Ann Oncol.

[42] Sankar K, Nagrath S, Ramnath N. Immunotherapy for ALK-Rearranged Non-small Cell Lung Cancer: challenges inform promising approaches. Cancers (Basel) 2021, 13(6). 10.3390/cancers13061476 PMC8004790 33806977

[43] Jahanzeb M, Lin HM, Pan X, Yin Y, Baumann P, Langer CJ (2021). Immunotherapy treatment patterns and outcomes among ALK-Positive patients with non-small-cell Lung Cancer. Clin Lung Cancer.

[44] Sholl LM (2022). Biomarkers of response to checkpoint inhibitors beyond PD-L1 in Lung cancer. Mod Pathol.

[45] Petitprez F, Meylan M, de Reynies A, Sautes-Fridman C, Fridman WH (2020). The Tumor Microenvironment in the response to Immune Checkpoint Blockade therapies. Front Immunol.

[46] Zeng C, Gao Y, Xiong J, Lu J, Yang J, Wang X (2020). Tumor-infiltrating CD8(+) T cells in ALK-positive Lung cancer are functionally impaired despite the absence of PD-L1 on Tumor cells. Lung Cancer.

[47] Jenkins MK, Khoruts A, Ingulli E (2001). In vivo activation of antigen-specific CD4 T cells. Annu Rev Immunol.

[48] Masopust D, Kaech SM, Wherry EJ, Ahmed R (2004). The role of programming in memory T-cell development. Curr Opin Immunol.

[49] Ohue Y, Nishikawa H (2019). Regulatory T (Treg) cells in cancer: can Treg cells be a new therapeutic target?. Cancer Sci.

[50] Xu F, Zhang H, Chen J, Lin L, Chen Y (2020). Immune signature of T follicular helper cells predicts clinical prognostic and therapeutic impact in lung squamous cell carcinoma. Int Immunopharmacol.

