# Supplementary figures and images for: Efficacy of immune checkpoint inhibitors in non-small cell lung cancer with NTRK family mutations
Source: BMC Pulm Med. 2023 Nov 29;23:482. doi: 10.1186/s12890-023-02707-x (PMC10688060; doi:10.1186/s12890-023-02707-x)

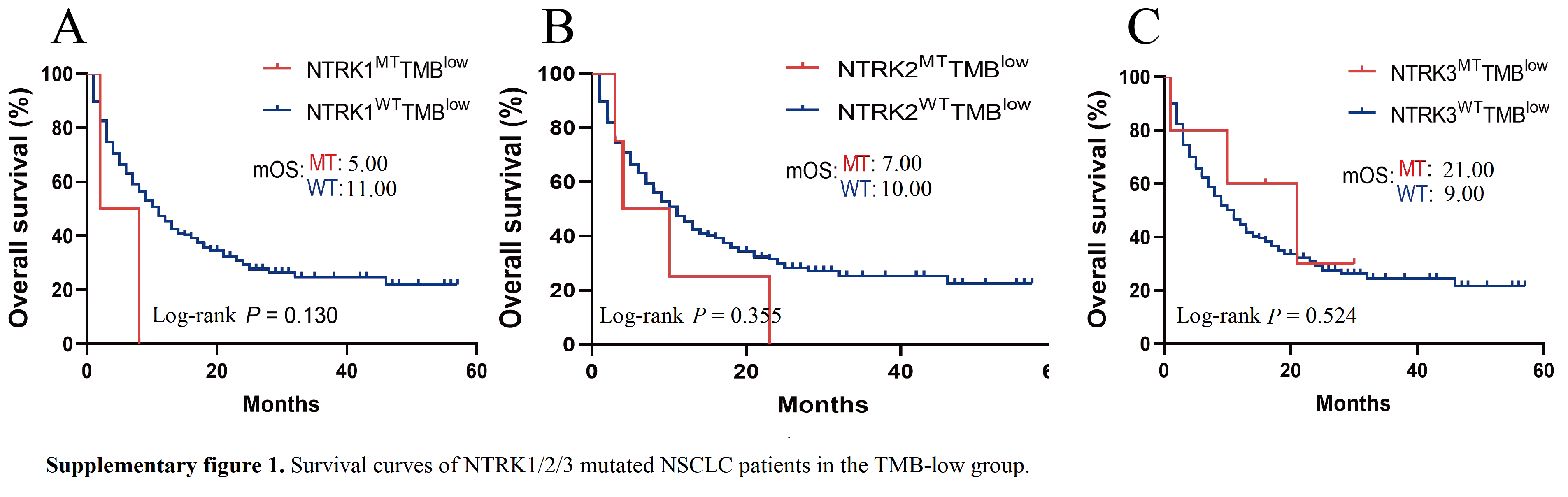

Supplement: Supplementary file 1 — Supplementary Material 1: Survival curves of NTRK1/2/3 mutated NSCLC patients in the TMB-low group [file 12890_2023_2707_MOESM1_ESM.png]

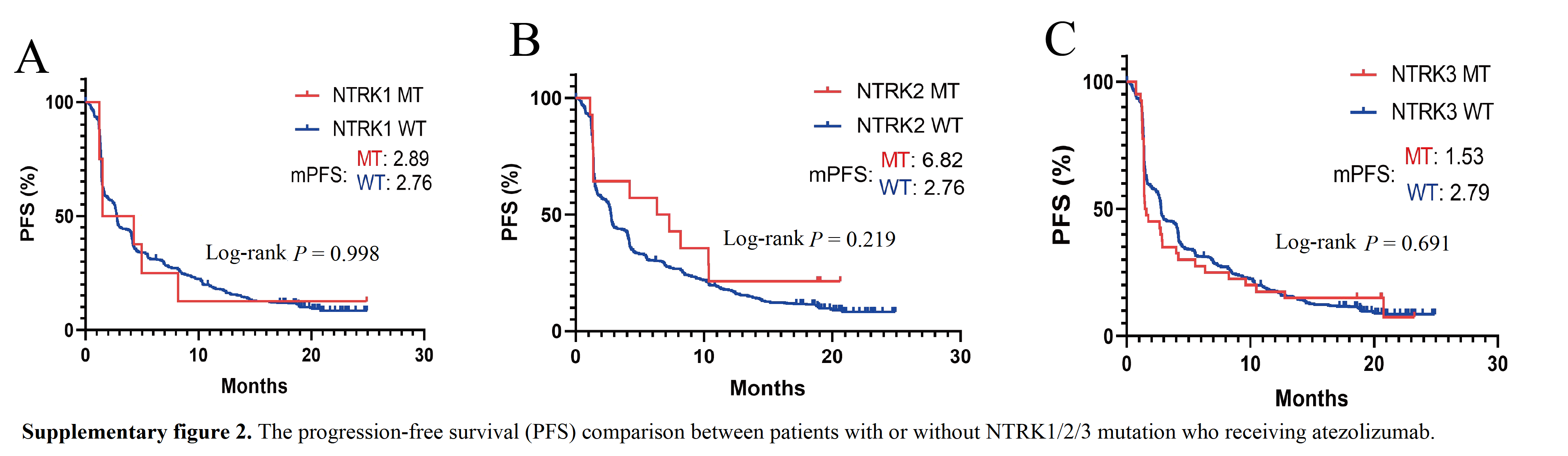

Supplement: Supplementary file 2 — Supplementary Material 2: The progression-free survival (PFS) comparison between patients with or without NTRK1/2/3 mutation who receiving atezolizumab [file 12890_2023_2707_MOESM2_ESM.png]

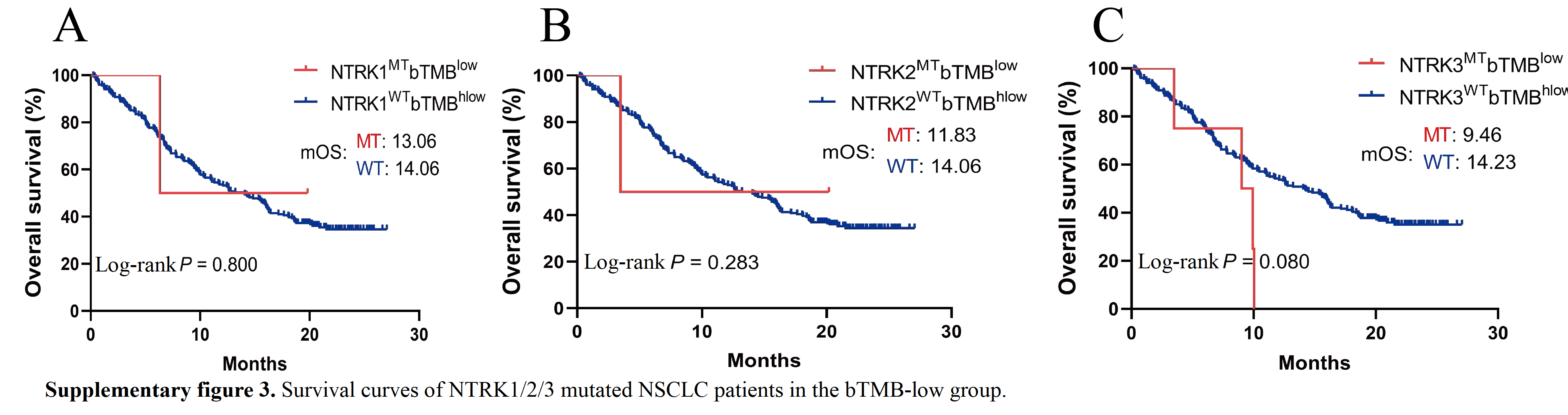

Supplement: Supplementary file 3 — Supplementary Material 3: Survival curves of NTRK1/2/3 mutated NSCLC patients in the bTMB-low group [file 12890_2023_2707_MOESM3_ESM.png]

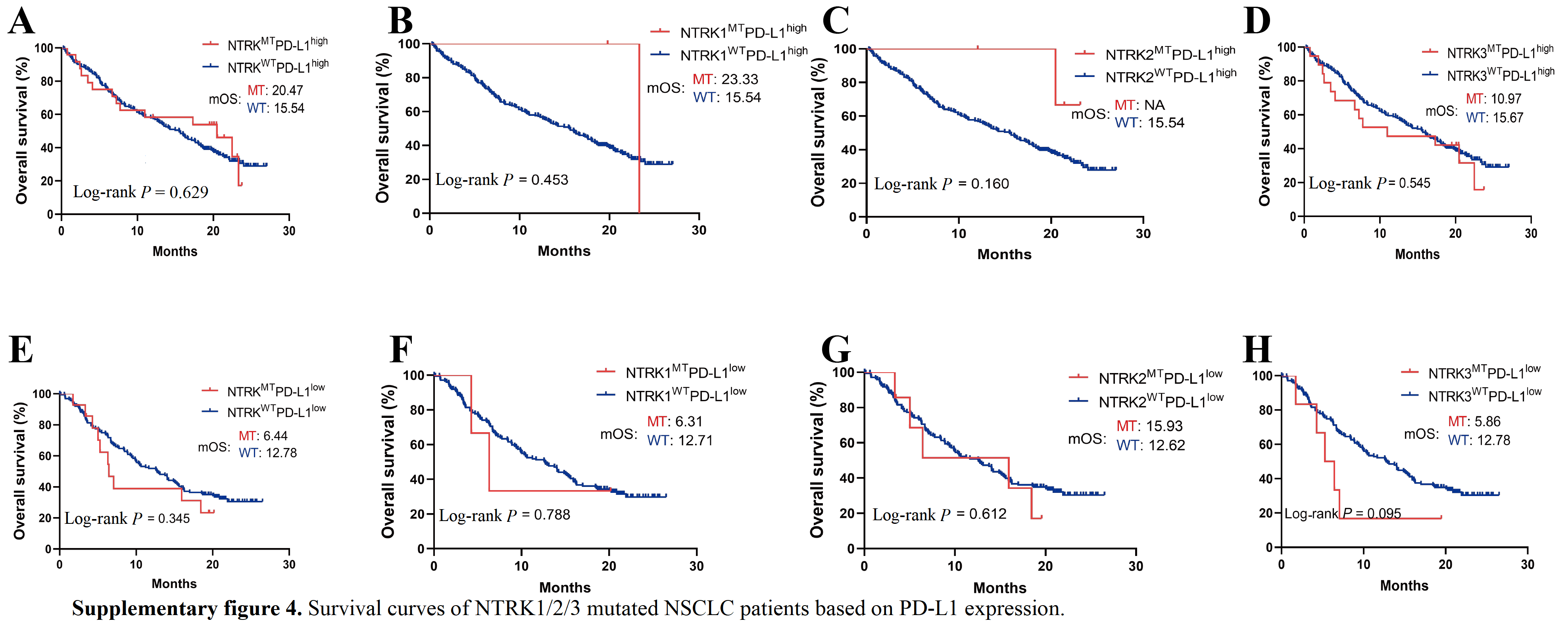

Supplement: Supplementary file 4 — Supplementary Material 4: Survival curves of NTRK1/2/3 mutated NSCLC patients based on PD-L1 expression [file 12890_2023_2707_MOESM4_ESM.png]

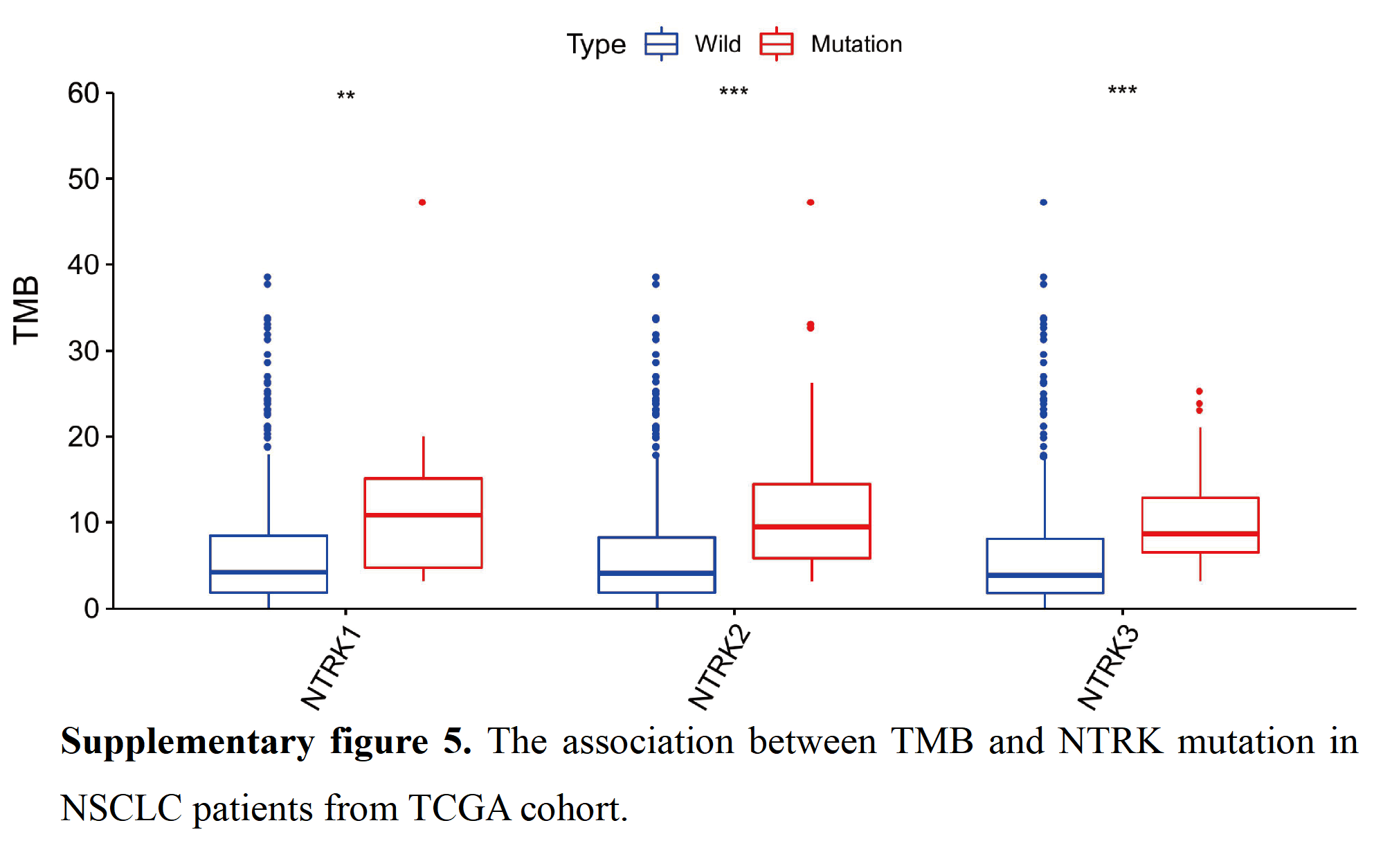

Supplement: Supplementary file 5 — Supplementary Material 5: The association between TMB and NTRK mutation in NSCLC patients from TCGA cohort [file 12890_2023_2707_MOESM5_ESM.png]

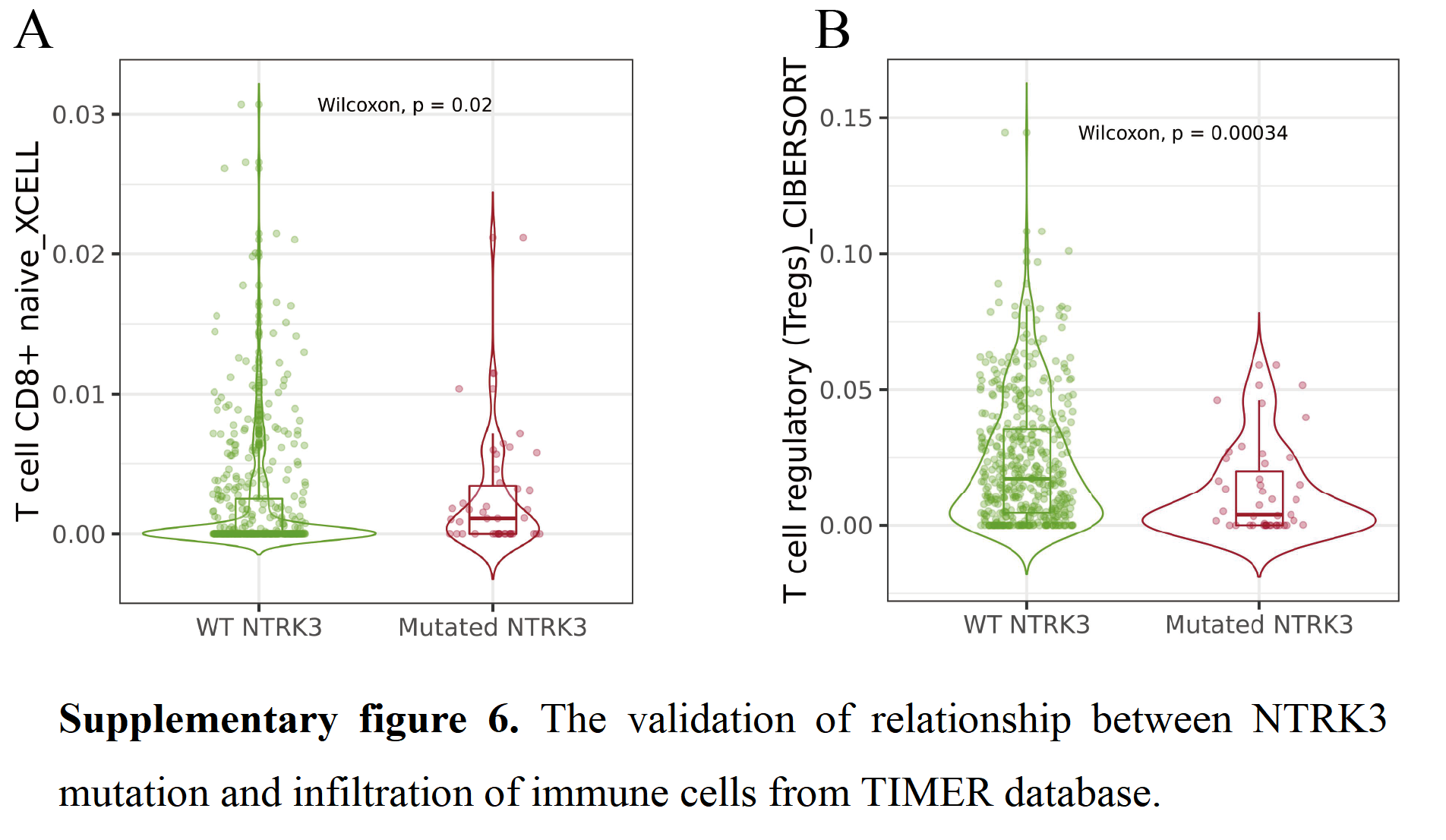

Supplement: Supplementary file 6 — Supplementary Material 6: The validation of relationship between NTRK3 mutation and infiltration of immune cells from TIMER database [file 12890_2023_2707_MOESM6_ESM.png]
